# Does ownership of improved dairy cow breeds improve child nutrition? A pathway analysis for Uganda

**DOI:** 10.1371/journal.pone.0187816

**Published:** 2017-11-10

**Authors:** Nassul S. Kabunga, Shibani Ghosh, Patrick Webb

**Affiliations:** 1 International Food Policy Research Institute (IFPRI), Kampala, Uganda; 2 Friedman School of Nutrition Science and Policy, Tufts University, Boston, MA, United States of America; University of Illinois, UNITED STATES

## Abstract

The promotion of livestock production is widely believed to support enhanced diet quality and child nutrition, but the empirical evidence for this causal linkage remains narrow and ambiguous. This study examines whether adoption of improved dairy cow breeds is linked to farm-level outcomes that translate into household-level benefits including improved child nutrition outcomes in Uganda. Using nationwide data from Uganda’s National Panel Survey, propensity score matching is used to create an unbiased counterfactual, based on observed characteristics, to assess the net impacts of improved dairy cow adoption. All estimates were tested for robustness and sensitivity to variations in observable and unobservable confounders. Results based on the matched samples showed that households adopting improved dairy cows significantly increased milk yield—by over 200% on average. This resulted in higher milk sales and milk intakes, demonstrating the potential of this agricultural technology to both integrate households into modern value chains and increase households’ access to animal source foods. Use of improved dairy cows increased household food expenditures by about 16%. Although undernutrition was widely prevalent in the study sample and in matched households, the adoption of improved dairy cows was associated with lower child stunting in adopter household. In scale terms, results also showed that holding larger farms tends to support adoption, but that this also stimulates the household’s ability to achieve gains from adoption, which can translate into enhanced nutrition.

## Introduction

The global burden of undernutrition remains high, particularly in Sub-Saharan Africa [1]. While a number of targeted nutrition-specific interventions are known to be effective in tackling undernutrition (such as iron and folate supplementation to pregnant women and promotion of exclusive breastfeeding), the international community continues to seek broader, so-called ‘nutrition sensitive’ actions that link investments in agriculture to improved child nutrition outcomes [2, 3]. Given that optimal animal source food consumption (ASF) is increasingly acknowledged as important to child growth, the promotion of livestock production is widely thought to support enhanced diet quality and child nutrition [4–9]. This can be achieved directly through own-consumption of nutrient-dense food, and/or indirectly by increasing income from sales that is used to purchase a more diversified diet [2, 10–13].

Uganda is a low-income country with high prevalence rates of child undernutrition. Recent data show that 29% of Ugandan children below age 5 were stunted (low height for age), with relatively higher stunting rates recorded in rural areas [14]. The combined crop and livestock subsectors employ over 69% of the population, with 25% of all households owning cattle [15]. In much of Uganda, dairy cattle are a dependable source of animal based foods and cash income through the sale of milk, cheese or yoghurt. Dairy cows also provide farmyard manure, important for the low-input smallholder integrated farming systems typical of East Africa. Moreover, dairy cows are some of the most important assets for Ugandan households, with potential for high asset-to-cash convertibility [16].

**Fig 1:**
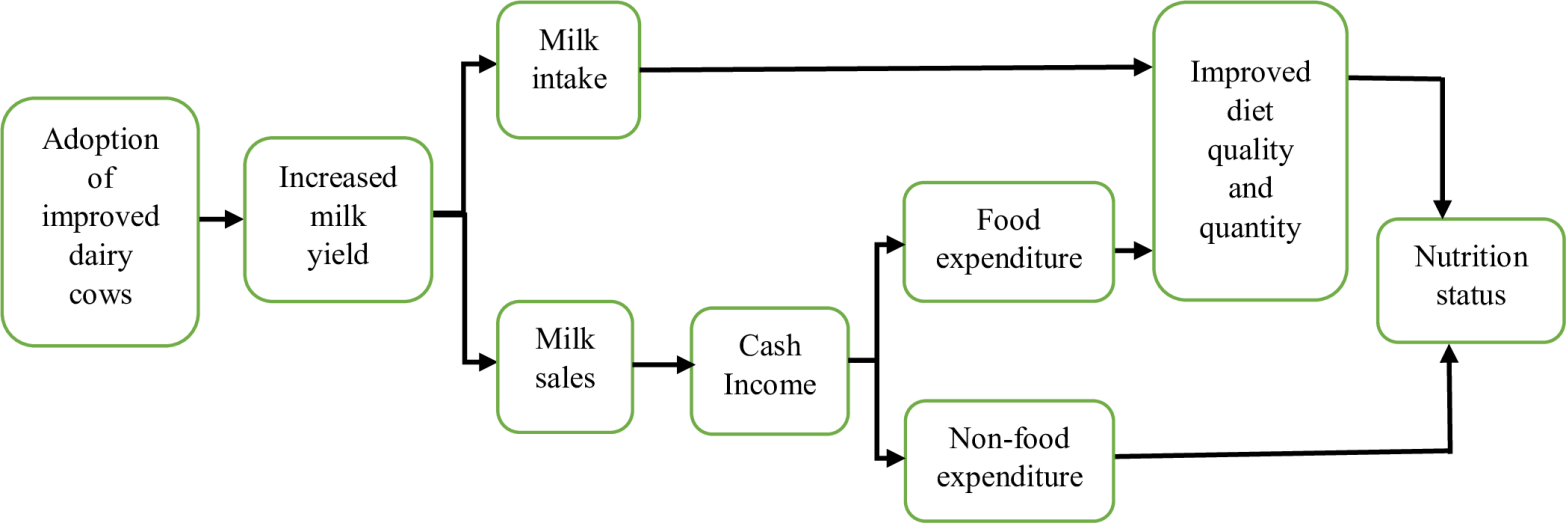
Simplified pathways between improved dairy cow adoption, welfare and child nutritional outcomes.

One of the major challenges to growth of Uganda’s dairy industry has been low productivity linked to low technology use, particularly with regard to use of indigenous cow breeds [17]. The government of Uganda alongside its development partners, have prioritized the promotion of dairy production to meet local and regional milk demands, owing to the favorable agro-ecological conditions in much of the country. The most significant step taken to date was the introduction of new dairy cow breeds in the 1990s and 2000s; these were of improved genetic potential (such as Holstein-Friesian, Guernsey, Jersey, and Ayrshire), carrying the potential of increasing milk production and productivity [17–19]. Nongovernmental organizations, including Send-A-Cow and Heifer International, have been particularly instrumental in promoting zero-grazing dairy cow units among women farmer groups [16]. Such a livestock-based intervention can leverage pathways to better nutrition, a causal pathway that has been demonstrated in earlier studies [10, 11, 20]. A simplified agriculture-nutrition pathway analysis is presented in Fig 1. Adoption of improved dairy cow breeds can increase milk production per unit area or per cow. Increased milk production may then catalyze increased milk intakes at the household level and stimulate rural milk markets, which helps integrate smallholders into modern value chains. Better producer incomes derived can then be used to purchase other food and non-food items to satisfy household nutritional needs.

Evidence on the impact of improved agricultural technologies in low-income countries is slowly growing, but often focusing on enterprise productivity, particularly through household income. The empirical evidence base linking agricultural strategies, including improved dairy cows adoption, to better health and nutrition outcomes, remains narrow, ambiguous, and based on a few heterogeneous studies that lack methodological rigor [2, 21–23]. For instance, some previous research demonstrates that cow ownership can enhance household welfare and individual nutrition outcomes: Nicholson et al. [24] found that ownership increased household-level intakes of dairy products as well as cash incomes in Kenya; while both Nicholson et al. [20] [using a sample of 370 households from coastal and highland regions of Kenya] and Rawlins et al. [25] [using a sample of 224 households from Northern Rwanda] indicated a marginally positive association between household ownership of dairy cows and child linear growth.

All these studies are site-specific, based on samples taken from areas with prior deliberate exposure to project activities. Without explicit consideration of differences in breed superiority and effectiveness of dissemination activities, these studies fail to draw and explain clear empirical pathways from adoption to welfare and child nutrition. Moreover, most studies presuppose that the benefits of adoption accrue uniformly across small and large farms, ignoring marginal benefits and costs due to scale.

This paper seeks to address such important gaps in the literature. Using a nationally representative sample of 907 Ugandan households, this study’s primary objective is to examine whether adoption of improved dairy cow breeds leads to enhanced child nutrition. Specifically, the analysis considers if differences in adoption status of improved dairy cow breeds are linked to farm-level outcomes (milk yield, own-milk intakes and milk sales) that manifest in household-level outcomes (food and non-food expenditure) as well as individual improvements in child nutrition (Z-scores for height-for-age, weight-for-age and weight-for-height).

## Methods

### Household survey

The data used for this study were derived from the 2009/2010 round of Uganda National Panel Survey (UNPS), conducted by the Uganda Bureau of Statistics (UBoS). The 2009/2010 round was the first wave of a panel of annual household surveys initiated to track changes in key household welfare indicators by ensuring improved quality and relevance of data on agriculture, food and nutrition security, among others. Moreover, the 2009/2010 also introduced a module on child and maternal anthropometry to assess changes in nutritional indicators. Unfortunately, the analysis for this paper is cross-sectional largely because subsequent data waves did not provide sufficient child anthropometric data observations for a feasible panel analysis.

The primary dataset comprised 2,975 households that were selected from the sample of households surveyed in the 2005/2006 round of the Uganda National Household Surveys (UNHS) following a two-stage stratified random sampling design [26]. In the first stage, enumeration areas were randomly selected from the four geographical regions based on probability proportional to size. Then, 10 households that had been randomly selected in the 2005/2006 were re-interviewed, except in cases where the respondent could not be traced. For this paper, the sample was further reduced to only 907 agricultural households that owned dairy cow enterprises. Of these, 745 households had complete anthropometric data for children aged below 5 years.

Data were collected between September 2009 and August 2010. To minimize recall biases and improve data quality, enumerators made 2 visits 6 months apart to all sampled agricultural households to capture agricultural indicators associated with two major cropping seasons in Uganda. The questionnaire recorded information on demography, assets, income, and expenditure as well as on agricultural production, including details on livestock production. Respondents were required to categorize different livestock breeds owned as either local, crossbreeds or exotic. Using this module, the analysis categorized households either as improved dairy cow adopters (subsequently, ‘*adopters’*) if they owned at least one crossbred or exotic dairy cow breed (of mainly, Holstein-Friesian, Guernsey, Jersey, or Ayrshire) or as ‘*non-adopters*’ if they reared only local indigenous dairy cows. To draw the empirical pathway linkages between improved dairy cow ownership and child nutrition, a broad set of potentially intermediate outcome indicators were created for this analysis.

### Derivation of outcome indicators

#### Farm and household level indicators

At the enterprise (or farm) level, annual milk yield per cow, estimated as total annual milk output divided by the number of cows owned, was used as an indicator of productivity. The share of milk sold, calculated as the ratio of the quantity of milk sales to total milk output and expressed as a percentage, proxies enterprise commercialization or milk market participation.

At the household level, milk intake and income were used to proxy for different dimensions of food and nutrition security. Annual per capita milk intake of own-produced milk, calculated as the annual milk reported consumed divided by the number of resident household members, was used as a marker of alternate access to ASFs, which serves as a proxy for micronutrient intake or diet quality. Caution should be taken while interpreting this proxy indicator, because it assumes equal distribution of milk among household members, which is seldom the case. Per capita expenditure (PCE) of food and non-food items was calculated by summing all household food and nonfood expenditures and dividing by the number of adult equivalents. Given the different recall periods used in the survey, conversion factors were applied to generate PCE on the basis of a 30-day month. Home-produced food consumption was valued at local market prices. PCE is a strong predictor for wellbeing and serves as a marker of food access for measuring gross food security [27].

#### Anthropometric measurements of children

The main objective of this study was to measure the extent of undernutrition in children aged 6 to 59 months across adopter and non-adopter households. That is, to test whether adoption status predicts nutrition outcomes in this cross-sectional sample. Anthropometric Z-scores, standardized for age and sex, were computed for height-for-age (HAZ), weight-for-age (WAZ), and weight-for-height (WHZ) using World Health Organization (WHO) Growth Standards [28]. Anthropometric Z-scores were calculated using a Stata-user written command [29]. Extreme values were removed as appropriate.

### Data analysis

Univariate analyses were performed using *t*-tests and Pearson’s chi-squared test of independence to first assess differences in socioeconomic, demographic and contextual characteristics between adopter and non-adopter households. Similarly, differences in the listed outcome indicators between adopters and non-adopters were assessed. Outcome indicators of interest were: i) annual milk yield to proxy for cow productivity; ii) milk sales as marker for household market participation or commercialization; iii) milk intakes and PCE, representing gross food and micronutrient security. Nutritional outcomes were iv) child anthropometric Z-scores as indicators of undernutrition.

With only cross-sectional data available, propensity score matching (PSM) methods were used to model the impact of improved dairy cow adoption on child nutrition outcomes and on hypothesized intermediary pathways. A few recent studies have used PSM to draw causal effects to child nutrition and health outcomes [25, 30, 31]. For this study, it was posited that despite initial investment costs for dairy cow systems, households that adopt improved dairy cows may increase milk yields, which in turn ensures increased on-farm per capita milk availability and intakes. Milk surpluses can then be marketed for cash, which may also lower milk prices for buying households. These effects may also potentially translate into significant increases in incomes of milk-producing households and ultimately may have broader implications for food security and the nutritional status of their members.

The use of PSM is justifiable due to potential non-random self-selection into adoption or non-adoption, which may result in biased estimates. PSM constructs a statistical comparison group based on a model of the probability of participating in the treatment (improved dairy cow adoption): *P*(*X*) = Pr(*D*_*i*_ = 1|*x*_*i*_). A propensity score *p*(*x*_*i*_) is obtained using a probit model which estimates the probability of participating in a treatment given a set of relevant observables, *x*_*i*_, likely to be correlated with participation in treatment, and with the outcomes of interest. *D*_*i*_ = 1 denotes treatment [32]. Adopters without appropriate matches from the non-adopter category (and vice versa) are dropped from further analysis.

Implementation of PSM requires a region of common support or substantial overlap in covariates between adopters and non-adopters; and that the balancing property is satisfied so that households being compared have a common probability of both being adopter and non-adopter [32]. Matching estimators were implemented using nearest neighbor matching (NNM) and tested for robustness using kernel based matching (KBM) algorithms [33]. All matching was done with replacement to increase the quality of the matches and reduce the chances of bad matches. Standard errors of the impact estimates were bootstrapped using 200 replications. The difference in outcomes between adopter and non-adopter households that have similar propensity scores (conditional on the set of observables) gives the average effect of the treatment (dairy cow adoption) on the treated (ATT).

PSM methods rely on observables to construct a comparison group. To rule out potential effects of unobservables, the analysis further conducted a battery of sensitivity analyses. First, bounding sensitivity method proposed by Rosenbaum [34] was used to test whether estimated results were sensitive to ‘hidden bias’ due to unobservables. Then, a method proposed by Ichino et al. [35] was implemented by simulating a set of ‘calibrated confounders’, which were then used as additional covariates in matching. Estimates earlier obtained (the ‘baseline’) were compared with and without matching on the ‘calibrated confounders’ to show how robust ‘baseline’ results are to unobservables constructed to reflect potential confounders. Finally, a different set of estimates were obtained using covariate matching methods following Abadie et al. [36]. Unlike PSM, covariate matching directly matches each adopter to the group of non-adopters with the smallest average difference in observables, determined by a multidimensional metric across all control variables.

A few limitations of the procedures used are noteworthy. Households were matched using cross-sectional data that does not indicate when a particular household was treated (i.e. adopted improved cows). While cautious in selecting covariates, the level of some covariates could have been determined after households had been treated. To cater for yet another source of potential confounding, all major models were re-estimated without all potentially problematic variables, particularly farm size and assets. Excluding these variables singly or together did not significantly affect neither the estimates nor the balancing properties.

A nationally representative dataset [26] was used for this analysis. It is not unexpected to find large variations in some observed characteristics. In particular, farm size and herd size, as some of the hypothesized drivers of our outcomes of interest, exhibited large standard deviations. To understand scale heterogeneity effects of adoption, further analysis compared impact results across two groups: small and large farms based on herd size and farm acreage. This was done by stratifying matched samples obtained using the NNM with all balancing properties satisfied, along the median.

Statistical significance was set at *P ≤* .*05*, and all data analyses were implemented using Stata software package, version 14.1.

## Results

### Descriptive analysis

Adopter households owned at least one crossbred or exotic (non-domestic) dairy cow in the year preceding the survey, while non-adopters owned indigenous dairy cows only. Table 1 shows that 21% of all Ugandan dairy cattle-herding households had adopted improved dairy cow breeds. The highest share of adopting 1households was in Western Uganda (38.1%) followed by Central Uganda (25.8%), then Eastern Uganda (16.3%) and the smallest share in Northern Uganda (2.2%). These observations are consistent to what was reported elsewhere: most dairy production takes place in Western Uganda. Central Uganda, where the capital city is located, provides the biggest market for raw liquid milk [16, 17].

**Table 1:** Number of sampled households and their adoption status by region.

	Central	Eastern	Northern	Western
Adopters	49	48	6	56
Non-adopters	141	246	270	91
Total	190	294	276	147

Table 2 presents summary statistics of household socioeconomic characteristics and of outcome indicators for the entire sample. Comparisons between adopter and non-adopter households were conducted because of the hypothesized difference in the impact of adoption improved dairy cows. Appropriate sample weights were applied so that the statistics reflect the characteristics in aggregate of all households in Uganda that are engaged in dairy cattle production.

**Table 2:** Summary statistics: farm and household characteristics, outcome indicators.

Characteristic	All sample			Adopters (*n* = 159)	Non-adopters (*n* = 748)
	N	Mean^a^	SE		
Education, head *(years)*	899	5.46	0.14	7.31 (4.34)	5.24 (4.17)***
Age, head *(years)*	906	48.38	0.50	50.09 (15.08)	48.04 (14.34)
Sex, head *(male = 1)*	907	0.77	0.01	0.79	0.77
Household size *(n)*	907	7.52	0.13	8.42 (3.96)	7.58 (3.48)***
Male share *(%)*	907	49.95	0.59	52.74	49.20**
Dependency ratio *(%)*	892	142.25	3.82	118.43 (95.17)	154.75 (116.35)***
Farm size *(acres)*	876	9.82	1.00	20.33 (73.86)	8.47 (16.90)***
Assets *(million UGX)*^b^	907	1.13	0.12	2.74 (6.09)	0.90 (2.95)***
Off-farm income	907	0.98	0.01	0.96	0.99**
Central *(yes = 1)*	907	0.22	0.14	0.30	0.18***
West *(yes = 1)*	907	0.18	0.01	0.35	0.12***
Urban *(yes = 1)*	907	0.09	0.01	0.16	0.10**
Annual milk yield *(l/cow)*	906	64.71	17.60	198.07 (513.48)	45.48 (150.67)***
Annual milk sales *(%)*	907	12.30	0.87	70.73 (190.43)	23.01 (65.29)***
Annual milk intake *(l/person*)	907	31.33	3.09	30.00 (35.38)	8.74 (22.22)***
Food PCE *(*‘000 *UGX*, *monthly)*^b^	906	27.51	0.69	29.77 (17.07)	25.27 (19.16)***
Nonfood PCE *(‘*000 *UGX*, *monthly)*^b^	863	18.93	0.78	27.96 (32.02)	15.84 (17.42)***
HAZ	745	–1.44	0.07	–0.93	–1.48***
WAZ	742	–0.83	0.05	–0.51	–0.83***
WHZ	735	–0.05	0.04	0.04	–0.03

Notes: N/n = No. of observations; SE = standard errors; PCE = per capita expenditure; UGX = Ugandan shillings.

^a^ Population-weighted statistics are reported

^b^ 1US$ = UGX 2,014 in the period 2009/10

*** and ** indicate that mean differences between adopters and non-adopters are statistically significant at *P≤* .*05* and .*1*, respectively.

An average household head in dairy cattle-herding households attained below the statutory 7 years of primary education and is aged 48 years. Most households were male-headed, composed of 8 members. The share of dependents was almost 1.5 times more than the working-age members. Adopters were significantly more educated, with larger family size, composed of mostly males of working age.

Sampled households owned, on average, 10 acres of land. However, half of these were smallholders, owning below 2 acres. Average asset holdings (excluding housing and livestock) were in excess of Uganda Shillings (UGX) 1 million. Adopters owned more than twice as much land and threefold the value of assets as non-adopters, construing that adopters were significantly wealthier than non-adopters. The majority of households (98%) were engaged in off-farm income activities (e.g. formal employment, wage labor, trading, making and selling crafts, etc.), perhaps as a risk diversification strategy to offset farm and household cash needs [37].

The sample was relatively balanced, with 40% of households residing in dairy cattle-herding areas of Central and Western Uganda, with a significantly higher proportion of households adopting improved cow breeds in those locations, as would be expected. Irrespective of regional placement, cattle herding, like other agricultural activities, was majorly rural, with the share of adopters significantly higher in rural areas.

Summary statistics of outcome indicators are presented in the lower panel of Table 2. Annual milk yield of dairy cows in Uganda was estimated at 65 liters/cow, which is much lower than in neighboring Kenya, in rest of Africa, and in Europe [38]. Households’ participation in milk markets was also low: only 12% of milk produced was sold. Although the biggest share of milk was retained for household consumption, annual per capita intakes of own-produced milk was far much lower than the international standards, [39] at only 31 liters/person. Compared to non-adopters, households owning improved dairy cows significantly obtained more than fourfold the volume of annual milk per cow, were more likely to participate in milk markets and consumed more than twice as much of own-produced milk.

Sampled households spent a monthly average of UGX 27,507 on food items per capita, which was slightly higher than the average national food poverty line (UGX 26,232) at the time, but spent about UGX 19,000 on nonfood items (e.g. education, health, clothes, etc.). Adopters spent significantly more on food and nonfood items, which can impact quality of life.

With regards to anthropometry, most children aged 6 to 59 months in sample households were below the WHO reference population mean height and weight. Average HAZ of –1.44 indicated that sampled children were one-and-a-half standard deviations (SDs) shorter than the reference population, suggesting gross stunting and chronic undernutrition. Similarly, average WAZ of –0.83 implied that weight of sampled children was below the standard. Average WHZ, indicative of more recent short-term deprivation, was not as bad, although still below the WHO recommendation. Children living in adopter households were significantly better nourished than children in non-adopter households, based on HAZ and WAZ scores. It is noteworthy that the number of data points for child anthropometry was substantially than for other variables because: (a) some sampled households had no eligible child, prompting enumerators to skip this section; and (b) some observations were voluntarily omitted from the sample due to measurement error during data processing. Nonetheless, these estimations mimic the Uganda Demographic and Health Survey at the time (HAZ = -1.4; WAZ = -0.8; and WHZ = -0.0) [40].

### PSM impact of improved dairy cows

The nature of farm and household characteristics laid out in the previous section hints at a positive selection bias in adoption behavior; that is, households with larger farms, more income and higher education are more likely to adopt improved dairy cows, which could of course bias impacts relating to child nutrition. PSM models were therefore employed to match similar households and compare outcomes across adopters and non-adopters.

Preliminary diagnostic steps are necessary before interpreting PSM impact results: A probit model to generate propensity scores upon which to balance the observed characteristics across adopters and non-adopters was in harmony with literature [41, 42], with most variables showing the expected direction of relationship (Table 3). For instance, adoption was positively associated with a higher level of formal education, large farms and female-headship. The latter is an interesting finding, as it demonstrates the effect of recent efforts to promote zero-grazing units, which are largely managed by women. For brevity, diagnostics are presented for only two outcome indicators, but were consistent across other 6 outcomes.

**Table 3:** Probit estimation of the propensity score.

*Dependent variable is adoption 1/0*	Enterprise and household-level indicators, for example, milk yield		Individual child-level indicators, for example, HAZ	
	Coefficient (SE)	z-value	Coefficient (SE)	z-value
Education	0.06*** (0.01)	4.00	0.08*** (0.02)	4.17
Age	2.16E–03 (4.13E–03)	0.52	2.62E–03 (5.36E–03)	0.49
Sex	–0.30** (0.14)	–2.13	–0.37* (0.20)	–1.90
Household size	0.01 (0.02)	0.63	0.00 (0.02)	–0.09
Male share	4.80E–03 (3.42E–03)	1.40	4.49E–03 (4.30E–03)	1.04
Dependency ratio	–1.00E–03* (5.47E–04)	–1.83	–2.15E–03*** (6.79E–04)	–3.16
Farm size	4.60E–03** (2.14E–03)	2.15	3.76E–03*** (1.36E–03)	2.76
Assets	0.04** (0.02)	2.37	0.02 (0.02)	0.73
Off-farm income	–1.01*** (0.37)	–2.77	–0.78** (0.40)	–1.95
Central	0.63*** (0.14)	4.54	0.77*** (0.15)	5.12
West	0.97*** (0.14)	7.02	0.95*** (0.17)	5.56
Urban	**0.02 (0.18)**	**0.11**	0.13 (0.22)	0.61
Constant	–0.79* (0.47)	**–1.69**	–0.76 (0.53)	–1.42
*N*	***853***		*690*	
*LR χ*^*2*^ *(p>χ*^*2*^*)*	***134*.*09(0*.*00)***		*107*.*39(0*.*00)*	
*Pseudo-R*^*2*^	***0*.*17***		*0*.*17*	
*Log likelihood*	***–334*.*24***		*–261*.*97*	

Notes: HAZ = height-for-age z-scores; SE in parentheses are standard errors; *N* = No. of observations

***, **, and * indicate statistical significance at *P≤*.*01*, .*05*, and .*1*, respectively.

**Fig 2:**
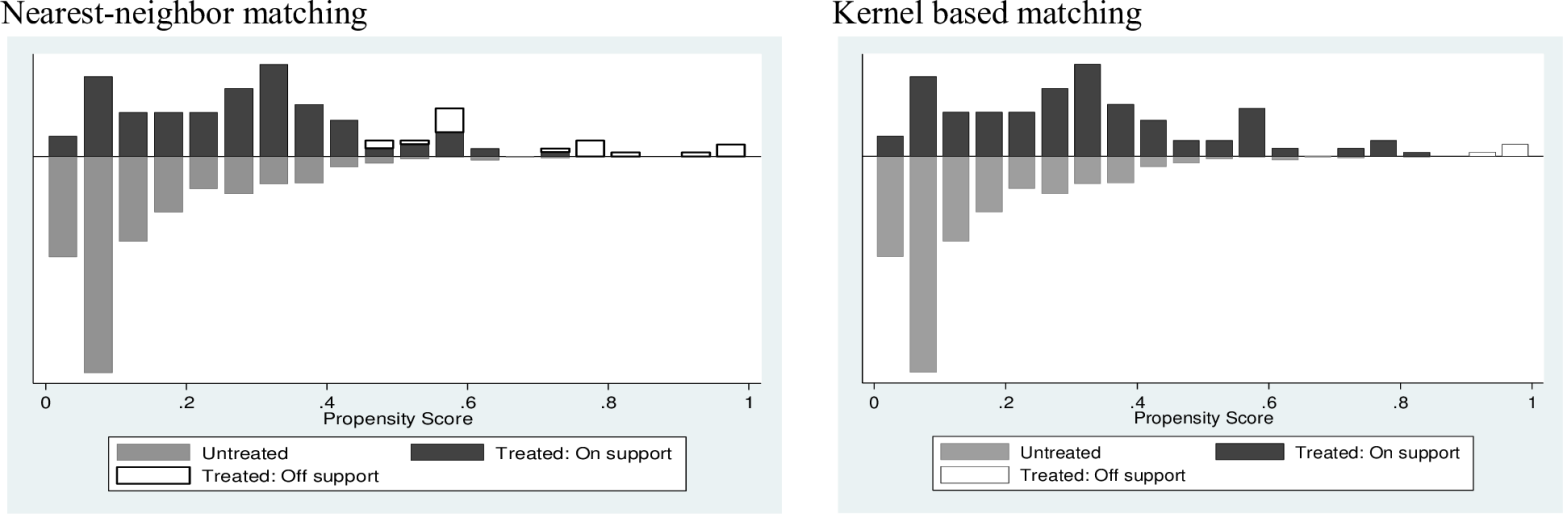
Propensity score distribution and common support: Impact on milk yield.

**Fig 3:**
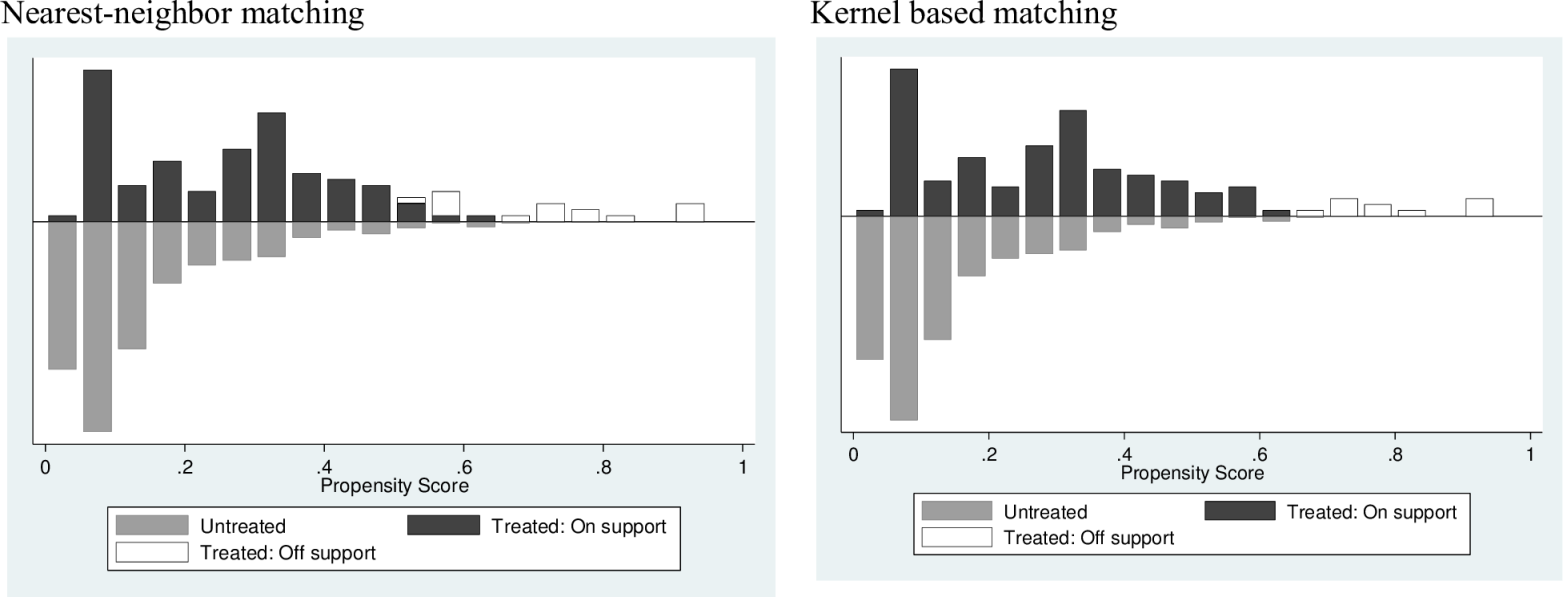
Propensity score distribution and common support: Impact on HAZ.

Figs 2 and 3 show that there was satisfactory overlap and the common support condition was satisfied. Finally, balancing tests showed substantial reduction in the mean absolute bias between the matched and unmatched models, with no significant differences after matching (Table 4). Before matching, many pairs of the covariate values appeared to be quite different. Matching brought them much closer.

**Table 4:** Covariate balance tests.

		Milk yield				HAZ			
		Mean		% bias reduction	*p-value*	Mean		% bias reduction	*p-value*
Variable		Treated	Control			Treated	Control		
Education	Unmatched	7.27	5.28	80.5	0.00	7.10	5.75	93.6	0.00
	Matched	6.95	7.34		0.46	7.13	7.04		0.84
Age	Unmatched	49.52	47.86	–2.4	0.18	46.35	44.12	35.6	0.10
	Matched	48.28	49.98		0.32	45.29	46.74		0.47
Sex	Unmatched	0.78	0.78	–985.0	0.96	0.85	0.88	–70.8	0.40
	Matched	0.79	0.77		0.66	0.86	0.91		0.27
Household size	Unmatched	8.55	7.75	91.6	0.01	10.09	9.09	77.8	0.01
	Matched	8.37	8.30		0.89	9.94	10.17		0.74
Male share	Unmatched	52.83	49.74	63.4	0.03	50.71	49.26	4.7	0.34
	Matched	52.63	51.50		0.59	50.32	51.70		0.45
Dependency ratio	Unmatched	116.97	156.84	96.9	0.00	149.67	193.32	88.8	0.00
	Matched	126.44	125.21		0.92	159.96	165.19		0.63
Farm size	Unmatched	20.67	8.56	71.9	0.00	29.36	8.40	98.4	0.00
	Matched	11.23	7.83		0.11	7.72	7.39		0.90
Assets	Unmatched	2.72	0.81	80.7	0.00	1.65	0.70	89.3	0.00
	Matched	1.69	1.33		0.33	1.08	1.18		0.73
Off-farm income	Unmatched	0.96	0.99	48.9	0.01	0.95	0.99	51.9	0.00
	Matched	0.97	0.99		0.41	0.99	0.97		0.32
Central	Unmatched	0.29	0.17	55.6	0.00	0.37	0.20	44.1	0.00
	Matched	0.29	0.24		0.33	0.37	0.27		0.14
West	Unmatched	0.35	0.12	77.7	0.00	0.27	0.09	51.1	0.00
	Matched	0.31	0.36		0.37	0.25	0.34		0.17
Urban	Unmatched	0.16	0.08	81.1	0.00	0.14	0.06	88.5	0.00
	Matched	0.14	0.13		0.72	0.10	0.11		0.82

Results for the net impact (ATT) of improved dairy cow adoption on outcomes are presented in Table 5. NNM estimates are interpreted and tested for robustness alongside the KBM algorithm. Adoption of improved dairy cows significantly increased annual milk yields by about 170 liters/cow, translating into more than 200% milk productivity increase. This is the average difference in milk yield between similar households that owned different cow breeds and consequently belong to different adoption status. Adoption also significantly and consistently increased own-milk intakes and milk sales by more than two-folds. Moreover, adoption significantly improved food PCE by about 16% but not necessarily nonfood PCE. Finally, although imprecisely estimated due to large standard errors, children under 5 living in adopter households were on average taller, attaining an additional 0.48 HAZ scores than children living in non-adopter households for the same age and gender. Adoption did not seem to influence WAZ or WHZ in any way.

**Table 5:** PSM results and sensitivity analysis.

Outcome		Mean		ATT (SE)	*t*-value	No. of observations		Critical level of hidden bias (Γ)
		Adopters	Non-adopters			Adopters	Non-adopters	
Milk yield	NNM	232.32	63.59	168.73*** (60.23)	2.80	149	700	1.40–1.50
	KBM	232.32	68.51	163.81*** (54.91)	2.98			1.20–1.30
Milk intake	NNM	66.20	27.04	39.16** (17.66)	2.22	149	700	1.20–1.30
	KBM	66.20	27.73	38.47** (16.86)	2.28			1.20–1.30
Milk sales	NNM	28.65	10.51	18.14*** (3.84)	4.73	149	700	1.50–1.60
	KBM	28.65	10.20	18.45*** (3.21)	5.74			1.60–1.70
Food PCE (log)	NNM	10.17	10.02	0.15** (0.08)	1.97	149	700	1.10–1.20
	KBM	10.17	10.03	0.14** (0.06)	2.52			1.40–1.50
Nonfood PCE (log)	NNM	9.71	9.58	0.13 (0.11)	1.14	145	668	—
	KBM	9.71	9.58	0.13* (0.07)	1.68			—
HAZ	NNM	–0.95	–1.43	0.48* (0.26)	1.81	108	572	1.25–1.30
	KBM	–0.95	–1.44	0.49** (0.22)	2.21			1.40–1.50
WAZ	NNM	–0.55	–0.65	0.10 (0.18)	0.55	108	569	—
	KBM	–0.55	–0.70	0.15 (0.14)	1.09			—
WHZ	NNM	0.01	0.11	–0.10 (0.18)	–0.58	102	564	—
	KBM	0.01	0.08	–0.07 (0.14)	–0.54	107		—

Notes: ATT = Average treatment effects; HAZ = height-for-age z-scores; WAZ = weight-for-age z-scores; WHZ = weight-for-height z-scores. NNN = nearest neighbor matching; KBM = kernel based matching.

***, **, and * indicate statistical significance at *P≤*.*01*, .*05*, and .*1*, respectively.

SE in parentheses are bootstrapped standard errors

### Sensitivity analysis

PSM constructs a comparison group based on a set of observables. To rule out potential effects of unobserved heterogeneity on ATT, a battery of sensitivity analyses were conducted. The treatment effect was robust to the possible presence of hidden bias, Γ, across all significant outcomes (Table 5). For instance, the value of 1.40–1.50 for milk yield implied that the credibility of a positive effect on milk yield would be questioned if households with similar characteristics differed in their odds of adoption by 40–50% [34]. Therefore, the level of hidden bias was substantially reduced by identifying the most important variables affecting both adoption and outcome indicators.

Following Ichino et al., [35] **S1 Appendix**, first row, shows that ‘baseline’ ATT estimates with no simulated confounder were very close to ATT estimates earlier attained (Table 5). Overall, estimates with simulated confounders conveyed the impression of robustness of the baseline ATT. For instance, if 74% of adopting households had attained more than average education, a potential confounder (mimicking education) would slightly increase the relative probability of increasing milk yields in case of no adoption (Γ = 1.21 > 1.00) and a much higher effect on the relative probability of adoption (Λ = 2.55 > 1.00), resulting in a new ATT = 158.5, which would be lower than the baseline ATT but nonetheless significant. The existence of a confounder that behaves like education could account for nearly 6% and 8% of the baseline impact estimate for milk yield and HAZ, respectively. **S2 Appendix** shows that ATT estimates following Abadie et al., [36] do not differ significantly from estimates in Table 5, further concretizing the robustness of the PSM results.

### Heterogeneous effects

Table 6 presents results of the analysis for heterogeneous effects of adoption. Ownership of improved dairy cows benefited both small and large farms, albeit disproportionately in favor of larger farms. In terms of herd size, higher impacts on milk yield, although imprecisely estimated (*p<0*.*1*), accrued to households with small farms of below midpoint (median) herd size (<2 heads). Small farms attained 56% more milk yield than households with large herd size. In contrast, farm acreage had a stronger leverage on milk yield than herd size: results showed that large-scale farmers, in terms of acreage, achieved much larger and highly significant productivity impacts from adoption of improved dairy cows. But in terms of both herd size and farm acreage, adoption of improved breeds significantly increased milk intakes, milk sales and child HAZ scores by relatively bigger magnitudes in large farms as compared to small farms. Farms of below median herd size and acreage were only significantly better with respect to expenditure on food items.

**Table 6:** Heterogeneous effects by scale: enterprise level.

		Herd size		Farm acreage	
		ATT (SE)	*t-*value	ATT (SE)	*t-*value
Milk yield	Large	118.66** (47.09)	2.52	311.52*** (108.22)	2.88
	Small	185.55* (112.00)	1.66	24.80 (32.92)	0.75
Milk intake	Large	64.98** (31.15)	2.08	89.02*** (33.89)	2.63
	Small	-0.29 (7.57)	-0.04	-5.17 (9.61)	-0.53
Milk sales	Large	18.68*** (5.39)	3.46	28.69*** (5.17)	5.55
	Small	10.78*** (3.92)	2.75	6.72* (3.81)	1.76
Food PCE (log)	Large	0.09 (0.09)	0.95	0.08 (0.09)	0.85
	Small	0.20*** (0.08)	2.64	0.21*** (0.06)	3.35
Nonfood PCE (log)	Large	0.12 (0.11)	1.11	0.14 (0.13)	1.11
	Small	0.11 (0.11)	1.04	0.08 (0.10)	0.78
HAZ	Large	0.86*** (0.32)	2.68	0.80** (0.32)	2.49
	Small	0.22 (0.32)	0.67	0.28 (0.29)	0.99

Notes: ATT = average treatment effects; SE in parentheses are bootstrapped standard errors

***, **, and * indicate statistical significance at *P≤*.*01*, .*05*, and .*1*, respectively.

## Discussion

Rigorous empirical studies linking agricultural interventions to nutrition outcomes remain limited. This analysis assessed the potential impact of genetically improved dairy cows on the nutrition of Ugandan children aged below 5 years, while drawing possible pathway linkages to intermediary farm and household outcomes. All impact estimates were tested for robustness and sensitivity to variations in observable and unobservable confounders. Moreover, we made more sense of the estimates by considering scale effects of adoption based on herd size and farm acreage.

At the enterprise level, we found that improved dairy cows increased milk yield of adopters by over 200%. These are substantial increases in milk yield, but still lower than the genetic potential of known improved breeds in Uganda and the region. For instance, previous research showed that an improved cow breed can produce over 990 liters/year compared to only 400 liters for local breeds [18], representing 148% increase in milk for improved breeds. In Kenya, a local zebu cow yields 100–200 liters/year yet an improved dairy breed yields 1,400–1,700 liters/year [38]. Thus, the potential to enhance milk productivity through adoption of improved dairy cows is irrefutable.

Improved dairy cows further enhanced the share of milk sales, an indicator of market participation, demonstrating the potential of improved agro-technologies in integrating smallholders into modern value chains. Unlike earlier studies, which reported ambiguity between commercialization and own-food consumption, [43, 44] this study showed that adopting improved dairy cows enhanced milk sales as well as own-produced milk intake, both of which could have wider implications for household welfare and nutrition outcomes.

At the household level, adoption of improved dairy cows significantly increased monthly food expenditures, but the positive impact on nonfood items was imprecisely estimated. The causal effect of adoption on monthly food PCE was about 16% relative to non-adopters, grossly implying that adopters were better off in terms of food access but not necessarily so in terms of nonfood items such as education, health, and clothing. Consequently, actual increased expenditures on food and potential enhancements in non-food expenditure may be attributed to increased milk intake and sales, which could then be reflected in leveraged child nutritional outcomes.

Finally, there were positive and consistently significant ATT estimates for HAZ scores, suggesting that children living in adopting households were on average taller than those living in non-adopting households for the same age and gender. However, improved dairy cow adoption did not influence WAZ or WHZ scores, implying that improved cow breeds reduced stunting but certainly not underweight or wasting of children below age 5 years. Although undernutrition was widely widespread in the original study sample and in matched households, the adoption of improved dairy cows was associated with augmented child height in adopter household by 0.48 SDs. These are quite large effects by any standards but are not surprising since milk is a known nutrient-dense ASF. Children who consume cow’s milk are known to attain higher height and bone mass than children who do not consume milk or other ASFs [5–8]. The biological mechanism between cow milk intake and child growth remains unclear but it is highly hypothesized that insulin-like growth factor 1 (IGF-1) contained in cow milk may contribute to cellular growth in bones and in other body tissues [5, 7].

Turning to scale effects, we found that adoption of improved dairy cows disproportionately benefited households with larger farms (both herd size and acreage). Except for food expenditures, whose adoption impact is more pronounced among smallholder farmers, households with large farms (>2 heads of cattle; > 2 acres of land) enjoyed greater adoption effects—in terms of milk yield, intakes and sales. However, farm acreage had a particularly stronger leverage on milk yield than herd size, which makes sense as it would be difficult to hold large herds without sufficient land. Seemingly, holding large farms is influential to adoption but also stimulates the household’s ability to achieve substantial gains from adoption, which may ultimately impact on child nutrition.

A few important caveats deserve mentioning. First, we emphasize that there are still few studies that rigorously evaluate the impact of agricultural interventions, drawing pathway linkages to household welfare and nutrition outcomes as was done in this case of improved dairy cows adoption in Uganda. Such an analysis is only possible if nutrition and health metrics are integrated into data collection exercises (as has been recently attempted in several countries), including in Uganda. Unfortunately, national-wide data collection systems, which integrate large agricultural and socioeconomic data together with nutrition metrics (e.g. physical growth indicators, blood biomarkers and cognitive performance) in a panel setup are still rare and, often not perfected over several data waves. Moreover, the ability to minimize sample attrition of these metrics in subsequent data collection rounds is essential for analysts to evaluate and design more effective nutrition-sensitive agricultural interventions.

Second, the heterogeneous analysis done for this paper teaches us that benefits of agricultural interventions do not necessarily nor uniformly accrue to all households. Had we not stratified the sample by herd size and farm acreage, the strong positive effects of adoption could have appeared to be uniformly distributed. Given sufficient sample size, other forms of stratification (e.g., based on gender, educational level, age, etc.) could provide more informative insights.

Third and most important, using our convenient choice of stratification, we learn that an agricultural intervention can possibly have different effects at different outcome levels. For this study, we found a disconnect between welfare indicators and child nutrition outcomes—improved dairy cows benefited larger farmers in terms of milk productivity, milk intakes and sales as well as reducing undernutrition but did not necessarily improve their household food security. Conversely, smallholder farmers improved their food expenditures due to adoption but this did not translate into improved nutritional outcomes. Such results suggest that the linkage between agricultural technology, welfare and child nutrition outcomes may not be straight forward, especially for the small farmers. There may be other factors to consider in future studies.

In particular, the results indicate that small farmers may have observed less than a minimal threshold required for the technology to leverage child nutritional outcomes. Since adoption stimulates milk sales to some level but does not enhance own-milk intakes for small farms, it is possible that additional income derived from the technology was not sufficiently used to buy more diverse foods or foods of improved quality but rather was spent on other goods that do not directly reduce child undernutrition.

However, it is also important to understand gender roles and intra-household food allocation dynamics, which may change in the face of the new technology [44, 45]. If women have less control of dairy cow enterprises, as was reported for Kenya’s milk-producing households, then child nutrition objectives may not be achieved [46]. The analysis of agriculture-nutrition linkages may often also be compounded by varying levels of nutritional knowledge among study participants, which is rarely captured in such data collection exercises. Our analysis was forced by the available data to assume equal distribution of milk intakes among household members (including children), did not delve into comparing dietary composition baskets across adopters and non-adopters and neither did it consider women roles and nutrition knowledge of study participants. This is an empirical weakness that could significantly affect estimates on child nutrition outcomes, which future studies should endeavor to address.

This research is important for Uganda and other countries in similar contexts. It shows how household-level agricultural interventions, specifically improved dairy cows, may help to ensure household food security and increase nutrient-dense ASFs, which in turn may support reduced stunting. This study therefore highlights the need to integrate nutritional metrics into traditional household surveys to allow analysts to better inform policy makers on such links. In addition, more rigorous impact and process evaluations are needed with panel data and possibly with randomization. Such studies should deliberately seek to identify key gaps along the impact pathway, which undeniably, is not well done for this study. The scope of this analysis was only limited to scale effects but future studies could analyze differential effects across other classifications, such as gender, education level, access to infrastructure and markets, among others. Such studies further help draw lessons for optimal targeting depending on need.

This study set out to rigorously test whether adoption of improved dairy cows can leverage pathways to improved child nutrition outcomes through enhanced milk productivity, milk intakes and milk sales. Our results show that improved dairy cows can indeed enhance household welfare and child nutrition. However, the analysis of agriculture-nutrition pathways still remains complex, requiring program implementers and researchers to take on a multifaceted approach. Without a comprehensive consideration of aspects such as agricultural and social-cultural norms and practices, gender roles, household resource and food allocation dynamics, dietary norms, among others, in programming for an intervention and in the research evaluation will likely lead to suboptimal policy choices.

## Supporting information

S1 Appendix Sensitivity analysis: Effect of “calibrated” confounders on outcomes. (DOCX)

S2 Appendix Covariate matching estimates following abadie et al. (36). (DOCX)
