# Supplementary material for: Does ownership of improved dairy cow breeds improve child nutrition? A pathway analysis for Uganda
Source: PLoS One. 2017 Nov 10;12(11):e0187816. doi: 10.1371/journal.pone.0187816 (PMC5681260; doi:10.1371/journal.pone.0187816)
Supplement: S1 Appendix — (DOCX) [file pone.0187816.s001.docx]

**Kabunga et al_Supplementary Information**

**S1 Appendix: Sensitivity Analysis: Effect of “Calibrated” Confounders on Outcomes**

|  | Milk Yield | | | | | | | |  | HAZ | | | | | | | |
| --- | --- | --- | --- | --- | --- | --- | --- | --- | --- | --- | --- | --- | --- | --- | --- | --- | --- |
|  | *p_11_* | *p_10_* | *p_01_* | *p_00_* | ATT | S.E. | Out. effect (Г) | Sel. effect (Л) |  | *p_11_* | *p_10_* | *p_01_* | *p_00_* | ATT | S.E. | Out. effect (Г) | Sel. effect (Л) |
| No confounder | 0.00 | 0.00 | 0.00 | 0.00 | 168.04*** | 55.63 | - | - |  | 0.00 | 0.00 | 0.00 | 0.00 | 0.47* | 0.25 | - | - |
| *Confounder like:* |  |  |  |  |  |  |  |  |  |  |  |  |  |  |  |  |  |
| Education | 0.74 | 0.68 | 0.53 | 0.49 | 158.53** | 63.32 | 1.21 | 2.55 |  | 0.71 | 0.67 | 0.52 | 0.51 | 0.51** | 0.21 | 1.05 | 2.28 |
| Age | 0.72 | 0.52 | 0.62 | 0.55 | 161.41*** | 62.13 | 1.40 | 1.17 |  | 0.52 | 0.44 | 0.50 | 0.30 | 0.45*** | 0.20 | 2.40 | 1.24 |
| Sex | 0.75 | 0.82 | 0.75 | 0.77 | 162.35*** | 61.81 | 0.94 | 1.21 |  | 0.79 | 0.83 | 0.78 | 0.85 | 0.47*** | 0.18 | 0.62 | 1.07 |
| Household size | 0.69 | 0.60 | 0.70 | 0.59 | 162.20*** | 61.72 | 1.69 | 1.10 |  | 0.46 | 0.46 | 0.38 | 0.38 | 0.48*** | 0.21 | 1.04 | 1.37 |
| Male share | 0.69 | 0.64 | 0.68 | 0.56 | 160.38*** | 62.10 | 1.73 | 1.41 |  | 0.68 | 0.46 | 0.57 | 0.55 | 0.47*** | 0.18 | 1.05 | 1.33 |
| Dependency ratio | 0.23 | 0.35 | 0.42 | 0.48 | 158.38** | 63.15 | 0.81 | 0.49 |  | 0.28 | 0.42 | 0.46 | 0.66 | 0.42** | 0.18 | 0.44 | 0.43 |
| Farm size | 0.51 | 0.26 | 0.34 | 0.39 | 161.70*** | 62.01 | 0.82 | 0.91 |  | 0.29 | 0.08 | 0.19 | 0.15 | 0.47** | 0.20 | 1.33 | 1.40 |
| Assets (million UGX) | 0.57 | 0.27 | 0.23 | 0.15 | 155.85** | 64.93 | 1.77 | 3.17 |  | 0.39 | 0.25 | 0.21 | 0.18 | 0.41** | 0.20 | 1.27 | 2.25 |
| Off-farm income | 0.99 | 0.95 | 0.99 | 0.99 | 160.42** | 62.37 | 1.03 | 0.38 |  | 0.96 | 0.92 | 0.99 | 0.98 | 0.45** | 0.21 | 2.12 | 0.24 |
| Central | 0.31 | 0.28 | 0.25 | 0.17 | 155.25** | 64.30 | 1.71 | 1.90 |  | 0.31 | 0.37 | 0.15 | 0.22 | 0.50** | 0.22 | 0.82 | 2.01 |
| West | 0.43 | 0.29 | 0.25 | 0.10 | 148.12** | 66.33 | 3.20 | 3.74 |  | 0.34 | 0.27 | 0.13 | 0.08 | 0.40* | 0.22 | 1.95 | 3.73 |
| Urban | 0.22 | 0.12 | 0.13 | 0.09 | 158.84** | 62.71 | 1.47 | 1.79 |  | 0.15 | 0.17 | 0.10 | 0.05 | 0.44** | 0.21 | 2.28 | 1.97 |

Notes: ATT = simulated average treatment effect, estimated by nearest neighbor propensity score matching, with 100 repetitions.

Г = average estimated odds ratio that the simulated potential confounder has on the probability of having a positive outcome;

Λ = average estimated odds ratio of the confounder on the probability of being selected for adoption;

S.E. = standard error

***, **, and * indicate statistical significance at the 1%, 5%, and 10% levels, respectively.
