# Supplementary material for: Does ownership of improved dairy cow breeds improve child nutrition? A pathway analysis for Uganda
Source: PLoS One. 2017 Nov 10;12(11):e0187816. doi: 10.1371/journal.pone.0187816 (PMC5681260; doi:10.1371/journal.pone.0187816)
Supplement: S2 Appendix — (DOCX) [file pone.0187816.s002.docx]

**Kabunga et al_Supplementary Information**

**S2 Appendix: Covariate Matching Estimates following**

**Abadie et al. (2004)**

|  | | ATT | S.E. | z-value |
| --- | --- | --- | --- | --- |
|  | *Enterprise-level indicators* | | | |
| Milk yield | | 164.80*** | 55.63 | 2.96 |
| Milk consumption | | 38.63** | 16.41 | 2.35 |
| Milk sales | | 18.66*** | 3.38 | 5.52 |
|  | *Household-level indicators* | | | |
| Meals | | 0.22*** | 0.07 | 3.27 |
| Food expenditure | | 0.16*** | 0.06 | 2.69 |
| Nonfood expenditure | | 0.17** | 0.08 | 2.18 |
| Food poverty – head count | | -0.13*** | 0.04 | -2.91 |
| Food poverty – gap | | -0.07*** | 0.02 | -2.92 |
| Food poverty – severity | | -0.04** | 0.01 | -2.42 |
| Nonfood poverty – head count | | -0.14*** | 0.05 | -2.66 |
| Nonfood poverty – gap | | -0.09*** | 0.03 | -3.08 |
| Nonfood poverty – severity | | -0.06*** | 0.02 | -3.04 |
|  | *Individual child indicators* | | | |
| HAZ | | 0.47** | 0.24 | 1.98 |

Notes: ATT = average treatment effects, corrected for bias;

S.E. = standard error, robust for heteroskedasticity around 2 nearest matches

*** and ** indicate statistical significance at the 1% and 5% levels, respectively.
